# A direct-from-serum method for calculating a type 1 diabetes genetic risk score

**DOI:** 10.1186/s12967-026-08930-4

**Published:** 2026-09-07

**Authors:** Benjamin Spurrier, Richard A. Oram, Nicholas J. Thomas, Jonathan M. Locke

**Affiliations:** Department of Clinical and Biomedical Sciences, Faculty of Health and Life Sciences, University of Exeter, Level 3, RILD Building, Barrack Road, Exeter, EX2 5DW UK

To the editor,

Type 1 Diabetes Genetic Risk Scores (T1D-GRS) have shown utility in diabetes prediction and classification [1]. For clinical implementation, assay costs must be minimised, yet standard protocols involving DNA purification, quantification, and storage add expense even before genotyping. Direct genotyping avoids these steps, offering a cheaper route to translation. T1D-GRS and islet autoantibodies are independent diabetes predictors [1]. Serum, used for islet antibody assays, contains sufficient extracellular DNA for SNP genotyping, dependent on a period of clotting permitting release of genomic DNA [2]. However, serum processing protocols sometimes have shorter clotting times (e.g. <30 min) that may be inadequate for release of gDNA into the serum [3]. This study assessed whether a 10-SNP T1D-GRS [1] could be accurately and efficiently calculated using a direct-from-serum genotyping method and how this was affected by serum clotting time.

All serum samples were separated from their clot by centrifugation after collection in serum separator tubes. Genomic DNA for comparison was purified from white blood cells using the Wizard Genomic DNA Purification Kit (Promega). TaqMan SNP genotyping assays and TaqPath ProAmp Master Mix (Applied Biosystems) were used to set up 5 µl qPCR reactions containing 1 µl purified DNA (~ 10 ng) or 1 µl serum. Plates included positive and no-template controls, were prepared on a Biomek i7 Automated Workstation, and run for 50 cycles on a QuantStudio 12 K Flex Real-Time PCR System. Genotypes were automatically called using Genotyping v4.1 app (Thermo Fisher Scientific).

We analysed a cohort (*n* = 116) of matched serum and whole-blood derived DNA samples received from across the UK and Ireland at Exeter Genomics Laboratory. Serum samples in this cohort had a clotting time > 24 h (median 5 days, range 1–14 days). After first-pass genotyping (i.e. no repeats), both biospecimens across all 10 SNPs had > 95% call rates (serum = 95–99%, purified DNA = 97–100%). Complete 10-SNP T1D-GRS was obtained in 98 (84.5%) serum samples verses 112 (96.6%) purified DNA samples (*p* = 0.003). In samples (*n* = 94) where both serum and purified DNA yielded a complete T1D-GRS, these were in 100% agreement (Fig. 1). In a subset of 32 samples with sequencing data, the serum-derived T1D-GRS was also 100% concordant.

**Fig. 1:**
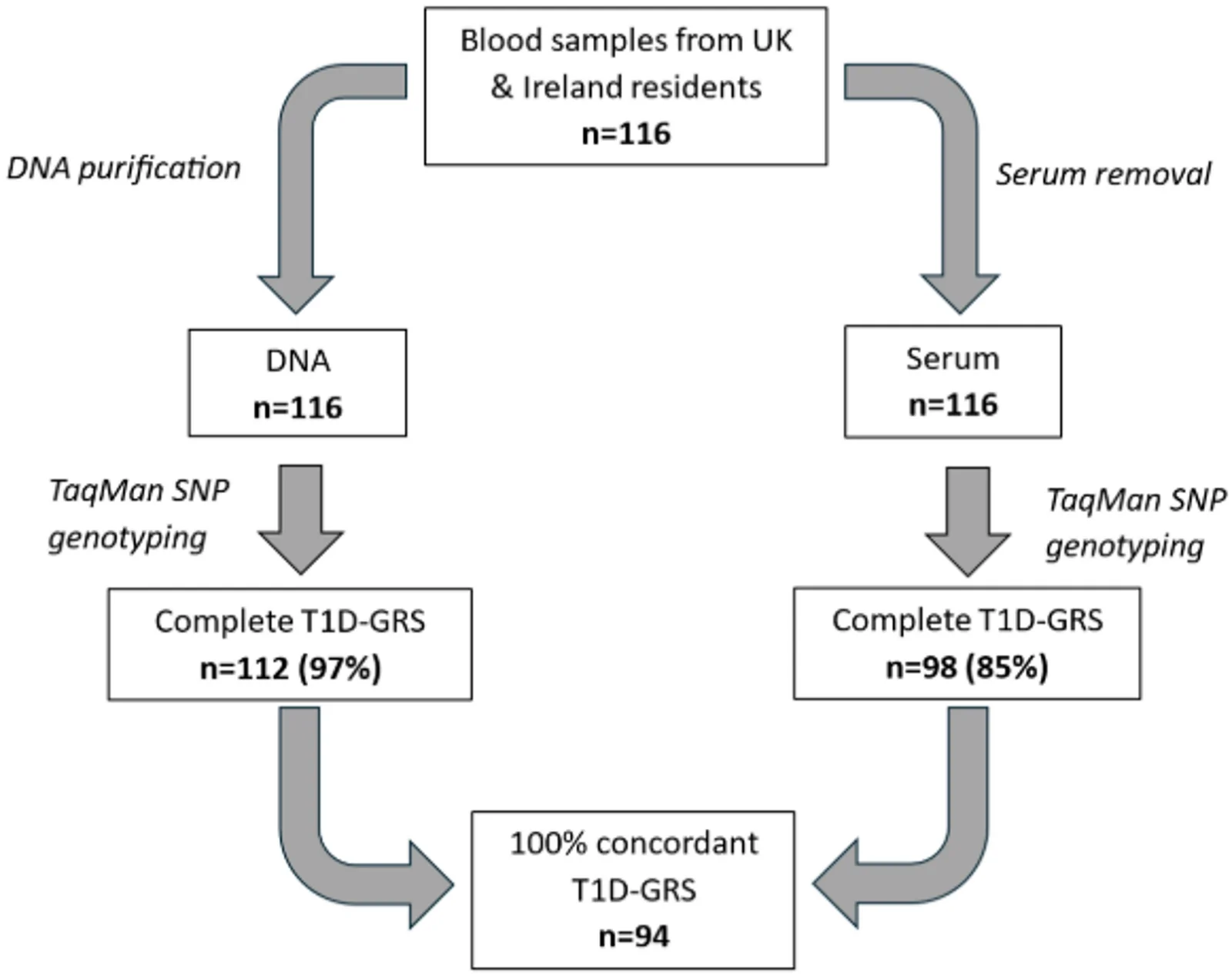
Efficiency and accuracy of calculating a 10-SNP T1D-GRS using TaqMan SNP genotyping assays and either standard purified DNA or serum as input

Having validated assay accuracy, we sought to determine the impact of clotting time on genotyping results. We analysed a cohort of serum samples (*n* = 122) with recorded clotting times (range 12–475 min). These comprised of samples with precisely recorded clotting times (*n* = 31, median 88 min) alongside a larger group (*n* = 91) processed under a standard protocol specifying ≤ 30 min clotting time. All samples were run in duplicate on separate qPCR runs to check concordance. No genotype calls were possible for 13/1220 (1.9%) of reactions.

We split the cohort by a 30-minute clotting-time cut-off. Shorter clotting times (≤ 30 min, *n* = 95) produced a genotyping success rate of 68.8%, a replicate discordance rate of 28.8% and complete 10-SNP T1D-GRS in only 7.4% of samples. Longer clotting times (> 30 min, *n* = 27) achieved genotyping success rate of 97%, replicate discordance rate of 2.2% and complete 10-SNP T1D-GRS in 77.8% of samples (Mann Whitney U = 188.5, Z = 6.84, *r* = 0.61, *p* < 0.001). Performance in the > 30-minute cohort was similar to the > 24-hour cohort (both genotyping 97%, complete T1D-GRS 77.8% vs. 84.5%, *p* > 0.05).

We have shown that a direct-from-serum genotyping method can accurately calculate a T1D-GRS. Serum samples separated > 30 min post-venepuncture achieved high call rates, only marginally less efficient than those using purified DNA. Samples < 30 min showed considerably reduced success, suggesting insufficient clotting time reduces extracellular DNA available for amplification. This aligns with previous findings that PCR genotyping from very low (< 56 pg) DNA concentration samples is unreliable [4].

These findings highlight a practical consideration for laboratories wishing to combine genetic and biochemical testing. For combined GRS and autoantibody testing, serum should clot for over 30 min to ensure sufficient cellular DNA release. This duration does not compromise autoantibody assays. If additional assays, sensitive to proteolysis, glycolysis or other degradation are required, an intermediate clotting period, over 30 min but under ~ 3 h, may provide an optimal balance [5].

This direct-from-serum approach could support cost-effective genotyping in large frozen serum biobanks where no DNA is available. Despite the modest number of SNPs tested, the method’s low input volume (1 µl per SNP) suggests feasibility for larger genotyping panels or other genotyping methods.

This proof-of-principle result could catalyse the development of direct, automated assays for laboratories without DNA-extraction capability, allowing parallel antibody and genetic testing from a single serum sample and supporting cost-effective clinical implementation.

## Data Availability

The datasets used and/or analysed during the current study are available from the corresponding authors on reasonable request.
